# The Relationship between Environmental Tobacco Smoke Exposure and Cardiovascular Disease and the Potential Modifying Effect of Diet in a Prospective Cohort among American Indians: The Strong Heart Study

**DOI:** 10.3390/ijerph14050504

**Published:** 2017-05-09

**Authors:** Sarah Rajkumar, Amanda M. Fretts, Barbara V. Howard, Fawn Yeh, Maggie L. Clark

**Affiliations:** 1 Department of Environmental and Radiological Health Sciences, Colorado State University, Fort Collins, CO 80523, USA; sarah.rajkumar@colostate.edu; 2 Department of Epidemiology, University of Washington, Seattle, WA 98195, USA; amfretts@u.washington.edu; 3 Medstar Health Research Institute, Hyattsville, MD 20782, USA; Barbara.V.Howard@medstar.net; 4 Georgetown/Howard Universities Center for Clinical and Translational Research, Washington, DC 20007, USA; 5 Center for American Indian Health Research, University of Oklahoma Health Sciences Center, Oklahoma City, OK 73190, USA; fawn-yeh@ouhsc.edu

**Keywords:** environmental tobacco smoke, cardiovascular disease, American Indian population, dietary effect modification, prospective cohort study

## Abstract

American Indians experience high rates of cardiovascular diseases (CVD). Environmental tobacco smoke (ETS) has been linked to CVD, possibly due to pro-inflammatory and oxidative stress pathways. We examined the relationship between self-reported exposure to ETS and fatal and nonfatal CVD incidence using Cox proportional hazards models among 1843 non-smoking American Indians participating in the Strong Heart Study. We also evaluated potential modifying effects of several dietary nutrients high in anti-inflammatory and anti-oxidant properties with ETS exposure on fatal and nonfatal CVD by creating interaction terms between ETS exposure and the dietary variable. Participants exposed to ETS had a higher hazard (hazard ratio: 1.22; 95% confidence interval, 1.03 to 1.44) for developing CVD compared to persons not exposed. Interaction analyses suggested stronger effects of ETS on CVD incidence among those consuming diets lower in vitamin E as compared to those consuming higher amounts, particularly on the additive scale. Additional research is recommended to clarify whether public health prevention strategies should simultaneously target reductions in ETS exposures and improvements in diets that may exceed the expected benefits of targeting these risk factors separately.

## 1. Introduction

Exposure to environmental tobacco smoke (ETS) is one of the most important and common sources of indoor air pollution worldwide [1]. ETS includes secondhand and thirdhand smoke and comprises therefore any tobacco smoke exposure outside of active smoking [2]. Despite the fact that smoking in most public places is prohibited, 25.3% of nonsmokers in the U.S. had elevated cotinine levels as recently as 2011/2012 [3]. Cotinine levels were highest among individuals with lower socio-economic status, and racial/ethnic disparities in the prevalence of smoking exist and appear to be widening [4]. In particular, American Indians have the highest rates of tobacco use in the U.S., although they smoke fewer cigarettes per day on average than other racial/ethnic groups and smoking rates between tribes vary dramatically [5,6]. Deaths related to smoking in women and men above the age of 35 years are higher among American Indians than the white U.S. population [7]. 

In general, American Indian populations experience higher rates of cardiovascular disease (CVD) and CVD-related risk factors as compared to the U.S. population as a whole [8]. Eichner et al. (2010) [5] reported an elevated risk of nonfatal CVD for current smokers compared to non-smokers among American Indians. Current epidemiologic evidence indicates that exposure to ETS may increase risk of cardiovascular disease by 25–30%, although recent studies suggest this risk may be larger [9,10]. The effects of ETS on CVD are, on average, 80% to 90% as large as those from active smoking [11]. The uncertainty that remains surrounding the magnitude of the association of ETS on CVD incidence may be due to the potential differences across populations studied (e.g., individual lifestyle, community, and societal factors) [9,12]. Therefore, although a major priority of public health policymakers is the continued support of programs aimed to reduce smoking initiation and increase smoking cessation, there is also a need to identify factors that modify the effects of ETS exposure on CVD outcomes [9].

ETS contains a complex mixture of small, respirable toxic chemicals. PM_2.5_ (particulate matter less than 2.5 microns in diameter), one of the constituents of ETS, has been implicated in CVD development [13,14] by inducing pro-inflammatory responses through the generation of oxidative stress [15,16]. Diets are a source of compounds with anti-inflammatory and anti-oxidant properties [17,18] that may counteract the negative effects of exposure to ETS. While corroborating evidence exists for asthma [19] and total mortality [20], whether differences in dietary intakes modify the effects of air pollutants on cardiovascular disease has rarely been investigated. In a study among 52 older adults, daily supplementation of fish oil reduced effects of ambient fine particulate matter on super-oxide dismutase activity, plasma glutathione, and heart rate variability [21,22]. Additionally, there is evidence that a diet high in fiber may protect against the harmful effects of ETS exposure on the risk for coronary heart disease mortality among Chinese non-smokers in Singapore [23].

To date, no studies have examined the relationship of ETS exposures and CVD endpoints among American Indian populations or assessed whether dietary factors modify the association of ETS on CVD incidence in a U.S. population. Using data collected as part of the Strong Heart Study, we examined the effect of ETS exposures on CVD incidence (fatal and nonfatal) among American Indian populations and also investigated whether high consumption of certain dietary nutrients attenuated this association.

## 2. Materials and Methods

### 2.1. Study Design

The design of the Strong Heart Study has previously been described elsewhere [24,25,26]. Briefly, the Strong Heart Study is a National Institutes of Health-funded prospective study of cardiovascular morbidity and mortality and CVD risk factors among 13 American Indian tribes/communities living in Arizona, Oklahoma, and North and South Dakota. Phase I of the study, 1989–1991, included 4549 men and women aged 45 to 74 years. Demographics and detailed information on CVD risk factors (e.g., smoking status, weight status, blood pressure, serum lipid levels, and glucose tolerance) as well as exposure to ETS were collected at that time [27]. Four years later, Phase II of the study began among 88% of the surviving members of the cohort. All were invited to participate in the dietary component of the study, for which 96% consented.

### 2.2. Study Population

For the present analysis, we started with the original Phase I cohort of 4549 participants and excluded participants based on the following criteria: 1033 belonged to a tribe that withdrew its consent from publication, 251 participants had suffered a cardiovascular incident before the study began, 2 had no information on smoking status, 1254 were current smokers, 16 had missing information on ETS exposure, and 150 had missing information on either hypertension, diabetes, calculated low density lipoprotein (calculated LDL) or albuminuria. We included 1843 non-smoking, disease-free participants to examine ETS exposure and CVD outcomes. A total of 1462 participants had available dietary information and could be included in the analysis on dietary effect modification.

### 2.3. Data Collection

Exposure to ETS was collected in three study centers using a questionnaire and recorded as number of hours per day the participant was exposed to ETS. For the analysis, a binary variable was created dividing participants into being exposed any amount of time or not at all.

Dietary intakes were estimated by an interviewer-administered 24-h recall collected at the Strong Heart Study clinics as described in detail by Stang et al. (2005) [8]. Briefly, interviewers were trained and supervised by Indian Health Service dietitians and were equipped with dietary assessment aids to use during the interview. Aids included two- and three-dimensional models of foods commonly consumed by American Indian populations. Data quality control measures and assurances were employed [8]. Vitamin A intake was converted to micrograms of Retinol Equivalents (mcg RE), all other nutrients were expressed in either micrograms (mcg), milligrams (mg), or grams (g). Total dietary energy intake was calculated as kilocalories (kcal) [28,29]. To express diet in terms of nutrient densities, intakes per 1000 kcal/day were calculated.

The outcome variable was incident CVD (fatal and nonfatal) as described previously by Eichner et al. (2010) [5] and Lee et al. [27]. CVD comprised of myocardial infarction, coronary heart failure (CHF), coronary heart disease (CHD) and stroke. The latter two were additionally evaluated in separate analyses. Fatal CVD incidence only was examined separately, as well. Mortality surveillance involved the identification of deaths through obituaries, tribal and Indian Health Service hospital records, and by direct contact with participants’ families. Physicians in the Mortality Review Committee of the Strong Heart Study independently reviewed all materials related to fatal events in order to confirm the cause of death. Morbidity surveillance involved medical record (hospitalized or outpatient visits) abstraction by trained investigators of the Strong Heart Study. Physicians of the Morbidity Review Committee reviewed charts to determine the specific cardiovascular disease diagnosis.

### 2.4. Data Analysis

To estimate the relative risk (hazard ratios, HR) and 95% confidence intervals (CI’s) of ETS exposure on CVD incidence, we ran multivariable Cox proportional hazards regression models. Assumptions for proportionality over time were tested using Kaplan-Meier curves, by assessing Schoenfeld residuals, and by dividing the analysis into two time periods. *p*-values < 0.05 were considered statistically significant. Follow-up for each participant was calculated as time from the date of the interview (Phase I) to the date of CVD diagnosis/death or to the date of death of other causes, or to the end of the study (31 December 2008), whichever occurred first.

The following covariates were collected and included in the full statistical models: age, sex, former smoking status, renal impairment (albuminuria), systolic blood pressure, calculated LDL, and diabetes status. A more parsimonious reduced model included a subset of covariates: age, sex, former smoking status, and renal impairment (albuminuria). Micro-albuminuria was defined as urinary albumin with a creatinine ratio equal to or greater than 30 mg/g (30 ≤ ratio < 300), and macro-albuminuria as a ratio equal to or greater than 300 mg/g of creatinine (ratio ≥ 300). 

To evaluate the potential modifying effects of dietary factors (total vitamin A, vitamin C, total vitamin E activity, beta-carotene, total fiber, water soluble fiber, insoluble fiber, alcohol, energy intake and total polyunsaturated fatty acids) on the relationship between ETS exposure and CVD incidence, we created an interaction term between the dietary variable (lowest quartile of intake versus the second through fourth quartiles of intake) and ETS exposure (within the models described above). All interaction models involving dietary factors also adjusted for total caloric intake. Interaction on the multiplicative scale was assessed by evaluating the p-value for the interaction term in the model. Within this model, we compared the HR for ETS among those with high dietary nutrient consumption versus the HR for ETS among those with low dietary nutrient consumption. Interaction on the additive scale was assessed by evaluating the relative excess risk due to interaction (RERI). Within this model, we used the non ETS exposed category with low vitamin E intake and the non ETS exposed category with high polyunsaturated fatty acid intake as reference categories, in separate models [30]. Stata/IC 13.1 and SAS (version 9.3, SAS Institute Inc., Cary, NC, USA) were used.

### 2.5. Sensitivity Analyses

Study center (Arizona, Oklahoma, Dakota) was also considered in our models to account for potential non-independence among participants within center; however, including study center as a fixed effect may serve as a proxy for the measures of interest, potentially over-adjusting our models; therefore, our primary models were conducted without center as a fixed effect. We also evaluated exposure to ETS in three categories (i.e., no self-reported exposure, less than or equal to 4 h of exposure, and greater than 4 h of exposure). All interaction analyses were also conducted using nutrient densities. Additionally, we repeated analyses removing the first 2 years of follow-up to account for potential reverse causality if those with pre-diagnostic symptoms changed behaviors. Furthermore, users of nonsteroidal anti-inflammatory drugs (NSAIDs), former smokers, and persons with morbidities such as diabetes and renal disease were excluded in separate sensitivity analyses. 

The Strong Heart Study protocol was reviewed and approved by the IRBs of the participating institutions, the 13 participating tribes, and the Indian Health Service IRBs for the three geographic areas wherein the tribes reside. Each participant gave informed consent. 

## 3. Results

A total of 1843 participants (1158 (62.8%) women and 685 (37.2%) men) were included in the present analysis examining the association between ETS exposure and cardiovascular incidence (Table 1). A total of 43.5% of women and 68.0% of men were former smokers. At the conclusion of the study, on 31 December 2008, 1152 participants were alive; 691 had died, 211 from a CVD event and 480 from other causes. In total, 574 persons had suffered a CVD event, out of which 363 were nonfatal. The most common type of incident was CHD though several cases (*n* = 234) could not be assigned to a single category.

Self-reported intake of NSAIDs was higher in women (25.9%) than in men (15.2%). Women also reported a history of diabetes more often (33.7%) than men (25.7%) while hypertension history was similar in both sexes (39.8% in women, 41.3% in men). Dietary factors were mostly not correlated among each other with a few exceptions: vitamin A and Beta-carotene (Spearman’s ρ: 0.79), soluble and insoluble fiber (Spearman’s ρ: 0.82). Total polyunsaturated fatty acids and total vitamin E activity exhibited moderate correlation (Spearman’s ρ: 0.63).

Being exposed to ETS was related to a higher hazard ratio for overall CVD incidence both in the full model (HR = 1.21, 95% CI: 1.02 to 1.43) and in the reduced model (HR = 1.22, 95% CI: 1.03 to 1.44; Table 2). When including study center as a fixed effect in the model, this effect was attenuated and no longer statistically significant (HR = 1.06, 95% CI: 0.89 to 1.26, full model; HR = 1.10, 95% CI: 0.93 to 1.31, reduced model).

We calculated interaction terms of dietary nutrients and exposure to ETS to evaluate potential modifying effects with ETS on CVD incidence. Multiplicative interaction analysis of vitamin E intake suggested that the relationship between exposure to ETS and CVD incidence was larger among those in the lowest quartile of vitamin E consumption as compared to those in the second through fourth quartile of vitamin E consumption (Table 3). The same tendencies were observed when evaluating effect modification of polyunsaturated fatty acids on the association between ETS and CVD incidence (Table 3). These patterns were not observed when evaluating interaction between exposure to ETS and other dietary nutrients (total vitamin A, vitamin C, beta-carotene, total fiber, water soluble fiber, insoluble fiber, alcohol, and energy intake). If study center was included as a covariate (fixed effect) in the models, the interaction analyses were further attenuated and no longer suggestive for vitamin E (*p* for interaction = 0.34) or polyunsaturated fatty acid intake (*p* for interaction = 0.73). We observed positive additive interaction for Vitamin E intake, suggesting that a decrease in ETS exposure would have a larger effect in the group with low Vitamin E intake (RERI = 0.35, 95% CI: 0.18 to 0.46). For polyunsaturated fatty acids, we observed negative additive interaction suggesting that a decrease in ETS exposure would have a larger effect in the group with low intake of polyunsaturated fatty acids (RERI = −0.32, 95% CI: −0.87 to 0.07), but this result was not statistically significant (Table 3).

When using nutrient densities instead of grams/day, results did not change. Including ETS exposure in three categories (i.e., no self-reported exposure, less than or equal to 4 h of exposure, and greater than 4 h of exposure) did not influence the results as both categories with any exposure were very similar in regard to CVD risk. When excluding former smokers, associations between ETS and CVD were stronger. Sensitivity analyses excluding the first 2 years of follow-up, those reporting NSAID use or persons with comorbidities did not meaningfully change risk estimates. 

## 4. Discussion

We observed a harmful effect of ETS exposure on fatal and nonfatal cardiovascular disease incidence among the Strong Heart Study population. ETS exposure has been shown to promote cardiovascular diseases in many studies [11,31] but uncertainty remains in the size of the association and the mechanistic pathways are still unclear. PM_2.5_ is a major constituent of ETS and has been shown to be redox active and to induce pro-inflammatory responses through the generation of oxidative stress [15,16]. This could be directly relevant to the potential mechanistic pathway involving the ameliorating effects of factors such as higher consumption of anti-inflammatory and anti-oxidant foods and nutrients [32,33]. For example, vitamin E, a well-known antioxidant [34], has been shown to reduce cardiovascular mortality and atherosclerosis in combination with a low cholesterol diet [35]. Although it does not seem to improve the lipid profile, there is evidence that vitamin E increases antioxidant gene expression and antioxidant enzymes [36]. Similarly, supportive evidence has been provided for polyunsaturated fatty acids; reduced effects of particulate matter on heart rate variability or biomarkers of oxidative stress were observed among those randomized to receive daily supplementation of fish oil as a source of polyunsaturated fatty acids [21,22,37]. Furthermore, results from an in vitro study suggested that vitamin E and polyunsaturated fatty acids may ameliorate the inflammatory and oxidative stress response initiated after vascular endothelial cells were exposed to PM_2.5_ [38]. Our results evaluating the potential modifying effect of vitamin E and polyunsaturated fatty acids were not statistically significant on the multiplicative scale. However, for vitamin E, effect modification on the additive scale was statistically significant suggesting that an ETS public health intervention might have a larger impact among populations with low Vitamin E consumption. Patterns suggest that multiplicative and additive effect modification by diet may be worth further consideration in larger studies.

We present our primary statistical models without the study center variable included as a fixed effect due to the potential that this variable’s inclusion may result in an over adjustment of our results. The three study centers have different smoking rates and Eichner et al. (2010) [5] reported differences in the self-reported number of hours per day exposed to ETS by geographic center. We also note the limitation for our primary models to control for potential non-independence of observations within study centers, which typically would lead to artificially small standard errors. However, inclusion of study center as a fixed effect impacted the magnitude of the effect estimates; results were attenuated as compared to results without adjustment for study center, indicating the potential for over-adjustment. Further validation for this modeling approach is evident in that our ETS risk estimates from the primary models are consistent with the magnitude of effect presented by the Institute of Medicine [9].

These results are limited by the use of self-reported exposure to ETS, making it impossible to distinguish between secondhand and thirdhand smoke, as well as the use of a single 24-h recall to assess diet. ETS exposure may be confounded by socio economic factors that can also be related to CVD incidence rates. To determine ETS exposure more reliably, more accurate long-term measures such as NNAL [4-(*N*-nitrosomethylamino)-1-(3-pyridyl)-1-butanol], a sensitive and specific marker of ETS exposure with a half-life of about 3 weeks, and cotinine, the primary metabolite of nicotine are recommended. Furthermore, evaluation of subclinical cardiovascular disease endpoints (e.g., blood pressure, markers of inflammation, or arterial stiffness as well as indicators of diabetes and pre-diabetes) could provide more detailed insight into mechanistic pathways. Finally, assessment of diet via a food-frequency questionnaire may allow for more accurate information on long-term nutrient intake patterns.

Air pollutant exposures are universal and often unintentional, and in the case for ETS, require substantial behavioral changes that are exceedingly difficult to implement in individuals or populations. Recommending avoidance is not always feasible; therefore, identifying effect modifiers that considerably reduce the risk of disease remains a reasonable objective in public health research. Given the large amount of time spent indoors (~22 h/day for Americans), as well as the widening ethnic/racial disparities in rates of both ETS exposure and CVD-related health outcomes, there is tremendous public health relevance in understanding how long-term exposure to ETS increases risk of developing CVD and whether or not certain subgroups of the population are more susceptible. Modifying factors, such as diets, may be important targets in future interventions to complement anti-smoking campaigns.

## 5. Conclusions

The present analysis confirms the previously observed detrimental influence of ETS exposure on cardiovascular health. The Strong Heart Study population allows for the examination of a unique population with high smoking rates but typically low doses. Our results suggest that further research (among larger sample sizes and utilizing methods described above to reduce the potential for exposure misclassification) may be warranted to investigate whether consumption of diets high in certain nutrients may reduce these damaging effects. Further research is needed to clarify whether public health prevention strategies should simultaneously target both reductions in ETS exposures and improvements in diets to exceed the expected benefits of targeting these risk factors separately.

## Figures and Tables

**Table 1:** Characteristics of Strong Heart Study nonsmokers (*n* = 1843) by sex and exposure to Environmental tobacco smoke (ETS).

Characteristic	Women (*n* = 1158)		Men (*n* = 685)	
	ETS exposure		ETS exposure	
	None	any	None	any
N (%)	657 (56.7)	501 (43.3)	335 (48.9)	350 (51.1)
Age at first interview, years, mean (95% CI)	58.2 (57.5 to 58.8)	56.4 (55.7 to 57.1)	57.6 (56.7 to 58.5)	55.0 (54.2 to 55.8)
BMI, kg/m^2^, mean (95% CI)	31.8 (31.3 to 32.3)	31.7 (31.2 to 32.2)	30.3 (29.7 to 31.0)	31.0 (30.4 to 31.5)
Person-years of follow-up (95% CI)	14.0 (13.6 to 14.4)	13.7 ( 13.2 to 14.2)	11.9 (11.3 to 12.6)	12.7 (12.0 to 13.4)
Center				
Arizona, *n* (%)	173 (74.2)	60 (25.8)	79 (79.0)	21 (21.0)
Oklahoma, *n* (%)	306 (58.5)	217 (41.5)	151 (45.1)	184 (54.9)
South Dakota, *n* (%)	178 (44.3)	224 (55.7)	105 (42.0)	145 (58.0)
Users of NSAIDs, *n* (%)	157 (52.3)	143 (47.7)	42 (40.4)	62 (59.6)
History of Diabetes, *n* (%)	224 (57.4)	166 (42.6)	93 (52.8)	83 (47.2)
History of Hypertension, *n* (%)	265 (57.5)	196 (42.5)	135 (47.7)	148 (52.3)
CVD incidence, *n* (%)	183 (55.3)	148 (44.7)	121 (49.8)	122 (50.2)
Previous smokers, *n* (%)	263 (52.2)	241 (47.8)	215 (46.1)	251 (53.9)
Albuminuria				
Micro, *n* (%)	129 (62.9)	76 (37.1)	59 (56.2)	46 (43.8)
Macro, *n* (%)	59 (56.2)	46 (43.8)	27 (52.9)	24 (47.1)
Dietary mean intake	Women (*n* = 953)		Men (*n* = 509)	
	ETS exposure		ETS exposure	
	none	any	none	any
Vitamin A, mcg RE, mean (SD)	1076.9 (1399.1)	1012.7 (1696.5)	1103.4 (1410.2)	988.9 (1853.4)
Beta-Carotene, mcg, mean (SD)	3080.3 (5480.0)	2877.2 (5035.9)	3857.2 (7325.0)	2735.5 (4567.3)
Vitamin C, mg, mean (SD)	110.3 (124.9)	104.2 (114.1)	103.4 (130.9)	104.9 (122.5)
Alcohol, g, mean (SD)	0.2 (1.5)	0.4 (4.4)	1.1 (7.7)	1.7 (11.7)
Total fiber, g, mean (SD)	17.0 (10.5)	15.5 (9.6)	18.0 (10.6)	18.8 (14.1)
Insoluble fiber, g, mean (SD)	10.7 (7.5)	9.8 (6.6)	11.2 (7.5)	11.8 (10.0)
Water soluble fiber, g, mean (SD)	6.1 (3.5)	5.6 (3.4)	6.6 (3.8)	6.9 (4.8)
Total Vitamin E Activity, mg, mean (SD)	10.3 (12.0)	10.2 (13.9)	9.2 (10.8)	9.1 (9.4)
Total polyunsaturated fatty acids, g, mean (SD)	11.1 (7.1)	11.9 (8.9)	12.4 (7.3)	13.6 (9.3)
Dietary energy, kcal, mean (SD)	1691.3 (656.3)	1728.0 (757.1)	1954.1 (847.3)	2012.3 (885.9)

CI, confidence interval; CVD, cardiovascular disease; NSAIDs, Nonsteroidal anti-inflammatory drugs; SD, standard deviation; mcg, micrograms; mg, milligrams; g, grams; RE, Retinol Equivalents.

**Table 2:** Exposure to environmental tobacco smoke (ETS) and CVD incidence among nonsmokers, Strong Heart Study (*n* = 1843).

Model	ETS Exposure	CVD HR (95% CI)
Full model ^‡^	No	1 (ref)
	Yes	1.21 (1.02 to 1.43)
Reduced model ^§^	No	1 (ref)
	Yes	1.22 (1.03 to 1.44)

CVD, cardiovascular disease; CI, confidence interval; HR, hazard ratio; ^‡^ Adjusted for age, sex, smoking history, albuminuria, systolic blood pressure, calculated LDL, diabetes; ^§^ Adjusted for age, sex, smoking history, albuminuria.

**Table 3:** Measure of the effect of ETS exposure on CVD incidence by Vitamin E and polyunsaturated fatty acid intake, Strong Heart Study (*n* = 1462).

	ETS and Diet Exposure Categories	ETS HR (95% CI) Within Strata of Dietary Factor ^§^	Interaction HR (95% CI) *^,§^
High Vitamin E intake (Quartiles 2–4)			
	ETS exposure: no	1 (ref)	1 (ref)
	ETS exposure: yes	1.17 (0.93 to 1.46)	1.17 (0.93–1.46)
Low Vitamin E intake (Quartile 1)			
	ETS exposure: no	1 (ref)	0.89 (0.65–1.28)
	ETS exposure: yes	1.57 (1.10 to 2.25)	1.41 (1.04–1.92)
*p* for interaction ^‡^		0.16	
RERI (95% CI) ^‖^			0.35 (0.18–0.46)
Low polyunsaturated fatty acid intake (Quartile 1)			
	ETS exposure: no	1 (ref)	1 (ref)
	ETS exposure: yes	1.52 (1.06 to 2.16)	1.52 (1.06–2.16)
High polyunsaturated fatty acid intake (Quartiles 2–4)			
	ETS exposure: no	1 (ref)	1.08 (0.79–1.48)
	ETS exposure: yes	1.19 (0.95 to 1.48)	1.28 (0.93–1.78)
*p* for interaction ^‡^		0.25	
RERI (95% CI) ^‖^			−0.32 (−0.87–0.07)

^‡^ *p*-value for effect modification on the multiplicative scale (generated from the product term of each dietary factor and ETS exposure); ^‖^ measure of effect modification on the additive scale; CVD Cardiovascular disease; CI, confidence interval; HR, hazard ratio; RERI, relative excess risk due to interaction; * HR for multiplicative interaction defined using no ETS exposure and more health beneficial dietary status as the reference category; ^§^ Adjusted for age, sex, smoking history, albuminuria, total calorie intake.

## Abbreviations

CHD	Coronary Heart Disease
CHF	Coronary Heart Failure
CVD	Cardiovascular Disease
ETS	Environmental Tobacco Smoke
HR	Hazard Ratio
LDL	Low Density Lipoprotein
NSAIDs	Nonsteroidal Anti-inflammatory Drugs
PM	Particulate Matter
RERI	Relative Excess Risk due to Interaction
